# The Structure of the World Pharmaceutical Market: Prioritizing Iran’s Target Export Markets

**Published:** 2019

**Authors:** Hosein Shabaninejad, Hasan Yusefzadeh, Gholamhossein Mehralian, Bahlol Rahimi

**Affiliations:** a *Department of Health Services Management, School of Health Management and Information Sciences, Iran University of Medical Sciences, Tehran, Iran. *; b *Department of Public Heath, School of Health, Social Determinants of Health Research Center, Urmia Medical Sciences University, Urmia, Iran. *; c *Department of Pharma Management and Pharmacoeconomics, School of Pharmacy, Shahid Beheshti University of Medical Sciences, Tehran, Iran. *; d *Department of Health Information Technology, School of Allied Medical Sciences, Urmia Medical Sciences University, Urmia, Iran.*

**Keywords:** Pharmaceuticals, Concentration ratio, World market structure, Herfindahl index, Import and export, Target markets

## Abstract

Iran`s pharmaceutical products market has faced fluctuations over time. Suitable market selection is necessary for stability of pharmaceutical exports. This study aimed to determine the structure of the world pharmaceutical market and to identify the target of Iranian pharmaceutical export. T do so, concentration ratios and Herfindahl index were used to address the world pharmaceutical market from 2001 to 2012. Also, a composite index was used to identify the target markets of Iran’s pharmaceutical industry. The results showed that the export side of world pharmaceutical trade has shifted to open oligopoly, thereby decreasing the monopolistic power of exporters. The import side, however, follows monopolistic competition. It has been observed that the structure of Iran’s pharmaceutical export is shifting to open oligopoly; though, pharmaceutical importers from Iran have not been stable. Moreover, 27 countries were identified as target markets. Due to significant differences between the current and potential export destinations of Iran’s pharmaceutical products, exporters should choose suitable strategies in order to diversify export markets. Such mechanisms as setting preferential tariffs on the basis of bilateral agreements, following effective advertisment, and paying attention to global consumers’preferences can be used to develop Iran’s pharmaceutical export to target countries.

## Introduction

One of the most important strategies for economic development is to develop non-oil exports. In order to increase non-oil exports, it is imperative to identify products and production activities that can penetrate international markets. Unbalanced economic structures must be modified, advantageous export goods and potential export markets must be identified, and this process must be supported by governments (1).

Pharmaceutical industry is one of the industries that can significantly contribute to economic development and create added value (2). In many countries, this industry has evolved technologically and most pharmaceutical companies are very active in research and development (3). 

The important features of this industry are creating high added value products and significant employment (4). World pharmaceutical market is rapidly evolving. Iran’s pharmaceutical industry is regarded as an important and well-established industry with a special place in the Middle East. However, Iran’s share of the world pharmaceutical market is only 0.25 percent (5). 

The rate of return on Iran’s pharmaceutical industry is over 25 percent, which is higher than most domestic industries. It is almost on par with large pharmaceutical companies in terms of rate of return (6). Iran is among the top 20 countries with the highest medicine consumption and is the third in Asia after Japan and China (7). The prevalence of self-medication is about 3 times the world average, with pain killers and antibiotics being the most self-administered medicines (8). In addition to a population of 77 million people with high drug use, Iran is located near a market of 47 million people in the southern end of the Persian Gulf where under development of health care has created an exceptional investment opportunity (9).

The development of pharmaceutical exports can lead to the growth of this sector. This not only has a direct impact on pharmaceutical products, but also improves labor and capital productivity (10). Identification of target markets focuses on marketing efforts and affects how resources are used (11).

Given the potential for production and development of pharmaceutical products in Iran and the high demand for Iranian pharmaceutical products among its neighboring countries, it is necessary to examine the structure of the world pharmaceutical market in order to increase production and exports and access to world markets, and to prioritize target markets in order to inform producers, exporters, and policy makers (12).

Iran’s trade policies emphasizes on non-oil export development, and the results of this study can provide useful insights for policy-makers in the pharmaceutical industry. Lack of investment in marketing and transport facilities and poor knowledge of pharmaceutical markets have reduced Iran’s pharmaceutical exports (13).

This paper tries to find how the structure of world pharmaceutical production and trade (imports and exports) has changed in the years of 2001-2012, the structure of Iran’s pharmaceutical export, the countries that can be potential target markets for Iran’s pharmaceutical exports, and the needs fulfilled to develop pharmaceutical export from Iran to target markets.

To our knowledge there has been no research on the world pharmaceutical market structure or target market prioritization similar to this study, but there have been studies on other industries in Iran and other countries briefly discussed in the following section. 

Hosseini and Hooman (2007) examined the world market structure of date palm and prioritized the target markets of Iran’s exports. The results indicated the oligopolistic structure of the world palm date production and the increasing share of Iran from the world market. Finally, 29 countries were prioritized as target markets for Iran’s palm date exports (14).

Abedin and Asgari (2005) prioritized the potential target markets for honey export from Iran. The results showed that the main honey importers from Iran were Turkey, United Arab Emirates, Azerbaijan, Kuwait, Saudi Arabia, and Qatar; while Germany, Saudi Arabia, U.S., Japan, and Liberia were identified as potential targets (15).

Shahiki Tash (2013) used indicators such as concentration ratio, Herfindahl-Hirschman, Tideman-Hall, comprehensive concentration, entropy concentration, and the Hannah-Kay indexes to determine Iran’s automotive market structure in 1996, 2001, 2006, and 2011. The results of this study revealed a high concentration in Iran’s automotive industry in spite of the emergence of new firms in that industry and the increasing production (16).

Macit (2012) used concentration ratio and Herfindahl-Hirschman index to investigate concentration and competition in the Turkish banking sector with empirical evidence over the period 2005-2010. He found no significant change in the degree of concentration from 2005. The results also showed that the Turkish banking sector is characterized by monopolistic competition and the degree of competition has decreased over the studied period (17).

Kramarić and Kitić (2012) analyzed the market structure and the degree of concentration of insurance markets in new EU member states with several indices such as concentration ratio, Herfindahl-Hirschman index, and entropy index. The results showed that the level of concentration decreased in all the observed countries, while in some countries it remained very high (18).

## Experimental

*Market Structure*

Market structure lies somewhere between monopoly and perfect competition. These structures differ in various features such as the number of companies, degree of freedom of entry and exit, information, homogeneity of products, and economic benefits. Perfectly competitive or monopolistic markets are rare. Market structure represents organizational features of the market with which the relationship between market components can be determined. In fact, market structure is a set of variables that show the market’s pricing and competition (19).

The main organizational features of the market are concentration of sellers and buyers, entry conditions, and product differentiation. Seller concentration is determined by the number of sellers and their distribution. Buyer concentration refers to distribution of products among different buyers. Higher percentage of product purchased by a small number of buyers indicates that higher buyer concentration and producers will not be able to set and establish their own price. In an extreme situation called monopsony a large buyer controls a large proportion of the market (19).

Product differentiation (homogeneous and non-homogenous) is an important market structure variable. Buyers may prefer some products over others due to differences in quality, design and packaging, and/or reputation and validity. Entry condition indicates the difficulty or ease of entry into a market. If market entry is difficult, the firms may choose to cooperate and collude and adopt non-competitive behavior (20).

The number of firms and firm size (scale) are two very important market structure variables. A market with a small number of firms will most likely be monopolistic. Also a market with a large firm and a number of small firms is more likely to be monopolistic than a market with a few firms of more or less equal size (20).

There are various indices in applied economics for measuring market structure, but Concentration Ratio (CR) and Herfindahl-Hirschman Index (HHI) are the most precise and well-known market structure indicators, so these were used in this study. Concentration ratio is the sum of the market shares of the i largest firms in a market, where *i* is a small number. The most common concentration ratios are CR_4_ and CR_8_, which indicate the market share of the four and the eight largest firms. The largest firm concentration ratio is CR_1_, which indicates monopoly, while in competitive markets CR_1_, CR_4_, CR_8_, and CR_16_ are common ratios. Concentration ratio can show the extent of market control of the largest firms in the industry and to illustrate the degree to which an industry is oligopolistic (21).

The Herfindahl index, proposed by Orris C. Herfindahl (1989), is a measure of the size of firms in relation to the industry and an indicator of the amount of competition among them. It is calculated from the following formula:

$H=\sum_{i=1}^{N}s_{i}^{2}$

where s_i_ is the market share of firm i in the market, and N is the number of firms. In this paper the number of countries and their relative share from the market are placed in the formula. The Herfindahl index can range from 0 to 1.0, moving from a huge number of very small firms to a single monopolistic producer (22).

*Target Markets*

Target markets are a set of customers that a business aims its marketing efforts and its merchandise towards. A variety of marketing strategies can be used to penetrate target markets. An international market with stable and continuous growth in demand and proper economies of scale can be considered as a target market. Modern marketing requires an accurate definition of target markets to be successful, as it will lead to better advertising and sales. Accurate identification of target markets is an important fact for the success of direct marketing (23).

In order to determine the target markets for Iran’s pharmaceutical exports, those countries that already had close economic ties with Iran and meanwhile were pharmaceutical importers from the world market were identified. Next, four following indicators of Iran’s pharmaceutical exports were evaluated and target markets for Iran’s pharmaceutical products were introduced based on the composite index of target market.

Import demand size: Taking into consideration only import demand rates, the number of countries that can be seen as potential target markets is determined by the percentage of world pharmaceutical import demand they make.

Import demand Index: Target markets for Iran’s pharmaceutical exports are those with an import demand index more than 100 in 2012 (assuming that 2001 = 100).

Import for domestic consumption: This includes countries that import pharmaceutical products for domestic consumption.

Countries share of Iran’s exports: This includes countries with a small share of Iran’s pharmaceutical exports and a larger share of world pharmaceutical exports.

Composite index of target market: It implies countries that have the following three features: First, those countries encompass more than 0.1 percent of world pharmaceutical imports; Secondly, pharmaceuticals has imported for the purpose of domestic consumption and the share of exports to imports for the given country is about zero; Thirdly, the given country does not account for a significant share of Iran›s pharmaceutical export and its share of the world imports is far more than Iran’s pharmaceutical export share to that country.

The related information and data were obtained from Iran’s pharmaceutical Statistics, World Bank, and International Trade Center (24-26). 

## Results

*The structure of the world pharmaceutical market*

In this section, we calculate concentration ratios to provide insights into the degree of competition and monopoly in the world pharmaceutical market.

Concentration in the world market was calculated based on the supply side (export) and the demand side (import) variables. More specifically, the Herfindahl index and concentration ratios of 1, 4, 8, and 16 firm were used to measure the market concentration in 2001-2012. Accordingly, the structure of the world pharmaceutical market (monopoly, competitive, hard/soft oligopoly, or dominant firm) and its changes over the period 2001-2012 were examined.

Germany, U.S., England, France, Switzerland, Belgium, Ireland, Netherlands, and Italy were the largest pharmaceutical exporters in the world in the years 2001-2012. In the export side of the world pharmaceutical market concentration ratios of 14.68%, 45.64%, 73.79%, and 90.37% were obtained for the 1, 4, 8, and 16 firm respectively for 2001-2012. The Herfindahl index in the export side of the world pharmaceutical market was 0.079 during this period. The inverse Herfindahl index (IHI) was 12.61 as were shown in Table 1.

The 1, 4, 8, and 16 firm (country) concentration ratios in the export side of the world pharmaceutical market changed from 14.26%, 45.81%, 74.34%, and 91.24% in 2001 to 14.79%, 45.16%, 70.71%, and 87.88% in 2012. In addition, the Herfindahl index in the export side of the world market changed from 0.0793 in 2001 to 0.0747 in 2012. IHI increased from12.62 in 2001 to 13.39 in 2012.

In the years 2001-2012, U.S., Germany, England, Belgium, France, Switzerland, Italy, Japan, Spain, and the Netherlands were the largest importers from the world pharmaceutical market. In the period 2001-2012, the 1, 4, 8, and 16 firm (countries) concentration ratios in the import side of the world pharmaceutical were 13.75%, 40.29%, 58.97%, and 75.82% respectively. The Herfindahl index was 0.0576, and IHI was 17.50 in this period (Table 2).

Concentration ratios in the import side of the world pharmaceutical market changed from 13.73%, 35.14%, 55.10% and 73.24% in 2001 to 13.58%, 35.67%, 54.72%, and 73.51% in 2012, but these changes were not significant. Additionally, the Herfindahl index in the import side changed from 0.0499 in 2001 to 0.0497 in 2012, while IHI changed from 20.06 in 2001 to 20.11 in 2012.

*The structure of Iran›s pharmaceutical exports to its business partners*

Table 3 shows the structure of Iran’s pharmaceutical exports to its business partners in 2012. In this year, Afghanistan, Russia, Iraq, Pakistan, Ukraine, Syria, Yemen, and Tajikistan were the major pharmaceutical importers from Iran. 1, 4, 8 and 16 country concentration ratios were 42.03%, 68.05%, 82.32%, and 92.57% respectively, indicating that while Iran’s pharmaceutical products were exported to 70 countries, the top eight countries imported about 70% of these products. The Herfindahl index was 21.1% for 2012, indicating the monopoly of a few countries over Iran’s pharmaceutical market. These eight countries had an even share of Iran’s total pharmaceutical exports and thus the exports structure of Iran is a form of closed oligopoly.

Although a few countries have a monopoly over Iran’s pharmaceutical market, they are not stable clients. However, the number of countries that import pharmaceutical products from Iran is increasing and Iran’s pharmaceutical exports structure is shifting to open oligopoly. 

**Table 1:** The structure of world pharmaceutical exports and its changes in the years 2001-2012

**Year**	**Concentration ratios and its changes**						**Herfindahl index**			**Structure**
	**CR**_1_	**CR**_4_	**CR**_8_	**CR**_16_	**% change in CR**_4_	**Largest exporters**	**HI**	**IHI**	**%Change in HI**	
	**(%)**	**(%)**	**(%)**	**(%)**	**(%)**		**(0-1)**	**(Number)**	**(%)**	
2001	14.26	45.81	74.34	91.24	...	Germany, U.S., England, France, Switzerland, Belgium, Ireland, Italy	0.0793	12.62	...	Oligopoly
2002	14.3	44.34	76.25	92.56	-3.22	Belgium, Germany, England, France, Ireland, U.S., Switzerland, Italy	0.0833	12.01	5.08	Oligopoly
2003	13.63	45.21	75.52	92.58	1.96	Belgium, Germany, England, France, Switzerland, U.S., Ireland, Italy	0.0807	12.39	-3.14	Oligopoly
2004	14.321	46.16	76.064	92.231	2.112	Germany, Belgium, Switzerland, France, U.S., England, Ireland, Italy	0.0833	12.01	3.24	Oligopoly
2005	14.538	45.683	74.259	90.832	-1.033	Germany, Belgium, Switzerland, France, U.S., England, Ireland, Italy	0.0808	12.38	-3	Oligopoly
2006	14.753	45.959	72.963	90.478	0.604	Germany, Belgium, Switzerland, U.S., England, France, Ireland, Italy	0.07941	12.59	-1.72	Oligopoly
2007	15.516	46.595	72.798	90.256	1.382	Germany, Belgium, Switzerland, U.S., England, France, Ireland, Netherlands	0.0804	12.4329	1.2863	Oligopoly
2008	15.776	46.301	73.715	89.938	-0.63	Germany, Belgium, Switzerland, U.S., France, England, Netherlands, Ireland	0.0799	12.5191	-0.6887	Oligopoly
2009	14.833	46.154	74.48	89.74	-0.318	Germany, Belgium, Switzerland, U.S., France, England, Ireland, Netherlands	0.0789	12.6808	-1.2748	Oligopoly
2010	14.559	45.074	73.023	88.86	-2.339	Germany, Belgium, Switzerland, U.S., France, England, Ireland, Netherlands	0.0761	13.1471	-3.5473	Oligopoly
2011	14.919	45.262	71.422	87.852	0.417	Germany, Switzerland, Belgium, U.S., Ireland, England, France, Italy	0.0755	13.2467	-0.752	Oligopoly
2012	14.796	45.168	70.719	87.884	-0.209	Germany, Switzerland, Belgium, U.S., France, England, Ireland, Italy	0.0747	13.3913	-1.0796	Oligopoly
2001-12	14.684	45.643	73.797	90.371	...	...	0.0793	12.6174	...	Oligopoly

**Table 2:** The structure of world pharmaceutical imports and its changesin the years 2001-2012

**Year**	**Concentration ratios and their changes**						**Herfindahl index**			**Structure**
	**CR**_1_	**CR**_4_	**CR**_8_	**CR**_16_	**% change in CR**_4_	**Largestimporters**	**HI**	**IHI**	**%change in HI**	
	(%)	(%)	(%)	(%)	(%)		(0-1)	(Number)	(%)	
2001	13.73	35.14	55.1	73.24	...	U.S., Germany, England, Belgium, France, Switzerland, Italy, Japan	0.0499	20.06	...	Monopolistic Competition
2002	13.85	43.83	61.86	77.67	24.74	U.S., Belgium, Germany, England, France, Italy, Switzerland, Netherlands	0.0634	15.78	27.12	Monopolistic Competition
2003	14.53	43.84	62.08	77.69	0.02	U.S., Belgium, Germany, England, France, Italy, Switzerland, Spain	0.0642	15.58	1.24	Monopolistic Competition
2004	13.45	43.36	62.01	77.55	-1.1	U.S., Belgium, Germany, England, France, Italy, Switzerland, Netherlands	0.0631	15.84	-1.64	Monopolistic Competition
2005	13.55	43.22	61.46	77.53	-0.33	U.S., Belgium, Germany, England, France, Switzerland, Italy, Netherlands	0.063	15.88	-0.23	Monopolistic Competition
2006	14.34	42.32	60.76	76.98	-2.06	U.S., Belgium, Germany, England, France, Switzerland, Italy, Netherlands	0.0614	16.28	-2.44	Monopolistic Competition
2007	13.95	41.76	60.4	76.37	-1.34	U.S., Belgium, Germany, England, France, Switzerland, Italy, Netherlands	0.0601	16.65	-2.24	Monopolistic Competition
2008	13.04	39.88	58.62	75.26	-4.5	U.S., Germany, Belgium, Netherlands, France, England, Italy, Switzerland	0.0559	17.9	-6.95	Monopolistic Competition
2009	13.29	39.34	58.34	75.51	-1.36	U.S., Germany, Belgium, Netherlands, France, England, Italy, Switzerland	0.0554	18.05	-0.83	Monopolistic Competition
2010	13.81	38.34	57.03	75.08	-2.54	U.S., Germany, Belgium, Netherlands, France, England, Italy, Switzerland	0.0541	18.49	-2.38	Monopolistic Competition
2011	13.85	36.72	55.21	73.53	-4.2	U.S., Germany, Belgium, France, England, Italy, Switzerland, Japan	0.0514	19.46	-5	Monopolistic Competition
2012	13.58	35.67	54.72	73.51	-2.87	U.S., Germany, Belgium, England, France, Japan, Italy, Switzerland	0.0497	20.11	-3.24	Monopolistic Competition
2001-12	13.75	40.29	58.97	75.82	...	...	0.0576	17.5059	...	Monopolistic Competition

Concentration ratios in the import side of the world pharmaceutical market changed from 13.73%, 35.14%, 55.10% and 73.24% in 2001 to 13.58%, 35.67%, 54.72%, and 73.51%in 2012, but these changes were not significant. Additionally, the Herfindahl index in the import side changed from 0.0499 in 2001 to 0.0497 in 2012, while IHI changed from 20.06 in 2001 to 20.11 in 2012.

**Table 3:** The structure of Iran’s pharmaceutical exports to its business partners in 2012

**Concentration ratios **					**Herfindahl index**		**Structure**
**CR**_1_	**CR**_4_	**CR**_8_	**CR**_16_	**Largest importers**	**HI**	**IHI**	
**(%)**	**(%)**	**(%)**	**(%)**		**(0-1)**	**(Number)**	
42.03	68.05	82.32	92.57	Afghanistan, Russia, Iraq, Pakistan, Ukraine, Syria, Yemen, Tajikistan	0.211	4.72	**Closed oligopoly**

Although a few countries have a monopoly over Iran’s pharmaceutical market, they are not stable clients. However, the number of countries that import pharmaceutical products from Iran is increasing and Iran’s pharmaceutical exports structure is shifting to open oligopoly.

**Table 4:** Target markets for Iran’s pharmaceutical exports

**Row**	**Description**	**Composite index of target markets**
1	Iraq	86.33
2	Afghanistan	82.96
3	Pakistan	71.14
4	Syria	70.56
5	Armenia	68.09
6	United Arab Emirates	63.41
7	Tajikistan	60.18
8	Russia	58.77
9	Uzbekistan	55.29
10	Azerbaijan	55.11
11	India	54.12
12	Oman	53.63
13	Yemen	47.08
14	China	46.19
15	Georgia	42.36
16	Sudan	41.83
17	Turkey	39.16
18	Algeria	38.06
19	Indonesia	35.27
20	Somalia	35.9
21	Jordan	33.8
22	Ukraine	33.25
23	Saudi Arabia	32.56
24	Uganda	30.69
25	Lebanon	29.86
26	Czech Republic	27.12
27	Italy	25.66

*Target markets for Iran pharmaceutical exports*

To identify and prioritize the target markets for Iran’s pharmaceutical exports, target market composite index was used. This index expresses the size of imported demand, imports for domestic consumption and the share of countries` exports from Iran. These indicators were evaluated annual trade statistics from UN Comtrade (27) and International Trade Centre (ITC) databases for the period 2001-2012. Potential target markets for Iran’s pharmaceutical exports are listed in Table 4.

According to target market composite Index, 27 countries from 225 countries that have been imported pharmaceutical products from the world market (represented in the Table 4) are the most appropriate countries with the highest priorities for developing export market and should be considered carefully as a pharmaceutical target markets of Iran.

## Discussion

The Herfindahl index and concentration ratios showed that the export side of the world pharmaceutical market is evenly distributed among 15-20 countries. In other words, the structure of the export side is open oligopoly, with 4 countries having a monopoly over 45% of the market. The increase in concentration ratios over the studied period indicate that the monopolistic control of exporters over the world pharmaceutical market has decreased and the structure of the world market export is shifting to open oligopoly.

The results also showed that the import side of the world pharmaceutical market is evenly distributed among 15-20 countries. In other words, the structure of the import side of the world pharmaceutical market is monopolistic competition; that is, the number of competing countries are high and they do not have monopolistic control over more than 15% of the market. Concentration ratios did not change significantly over the studied period, indicating that importers have maintained their monopolistic control and the structure is still monopolistic competition. 

A comparison of the export and import side of the world pharmaceutical market suggests that the import side is more competitive than the export side. Thus, it is the importers and not the exporters that dominate the world pharmaceutical market.

Examining the structure of Iran’s pharmaceutical exports revealed that Iran’s major business partners changed over the studied period due to lack of stability in the market. However, recent increase in the number of importers from Iran’s pharmaceutical market has reduced the monopolistic power of Iran’s business partners.

Among the 225 countries that import from the world pharmaceutical market, there are at least 27 countries with appropriate distance and consumption pattern (Low-income developing countries) which are the best candidates to be considered as target markets for Iran’s pharmaceutical exports.

The major importers from Iran’s pharmaceutical market during the study period were Iraq, Afghanistan, and some member countries from the Common wealth of Independent States (CIS). However, this study identified Pakistan, Syria, Armenia, UAE, Tajikistan, Uzbekistan, Azerbaijan, and some other countries as potential target markets for Iran’s pharmaceutical exports. 

Pharmaceutical firms can choose a number of high-priority markets with respect to their capabilities and potentials, and penetrate those markets using an integrated marketing plan that focuses on information about competitors, the marketing mix, and relevant rules and regulations.

The results suggested that the potential markets for Iran’s pharmaceutical exports are mainly regional markets. In addition, some of these countries have low rankings on the list of priority markets. This does not mean that export to these markets should be limited; rather penetration into these markets should be accelerated with an accurate marketing plan given the growth in demand as well as the increasing potential for competition.

To achieve a proper place in the international pharmaceutical trade, the following strategies are recommended:

Diversifying target markets for exports;

Strengthening marketing and packaging infrastructure based on standards and consumer needs;

Facilitating export laws and eliminating shortcomings in product transportation;

Developing production and export organizations by emphasizing on production for export purposes;

Reinforcing trade associations (government support for facilitating export by creating integrated marketing associations, establishing sales offices in target countries, and holding pharmaceutical exhibitions in target countries);

Prioritizing target markets based on import demand size, import demand index, import for domestic consumption, and countries’ share of exports;

Developing exports to target countries through mechanisms such as preferential tariffs trade agreements, effective promotion, exhibitions, quality assurance, and attention to standard as well as consumer tastes and preferences (28);

Participation of pharmaceutical companies in exhibitions with financial support by the government;

Formation of holding companies in which case the composition and structure of pharmaceutical companies change and the companies will cooperate and not compete;

Solving the marketing and financing problems of small pharmaceutical companies through brand licensing;

Creating pharmaceutical export associations and unions.

## Conclusion

Prioritizing target markets of pharmaceutical export, based on the target market composite index, indicates Iraq and Afghanistan as the first priority to be considered as target countries for Iran pharmaceutical export. Pakistan, Syria, and other countries of target markets for Iran’s pharmaceutical exports take the next order in this list. Given the current position of Iran’s pharmaceutical export to different countries, there is a significant difference between actual and potential markets; this means that the potential target markets aren’t mainly the same as the current markets. Meanwhile, considering the lack of a classified and comprehensive marketing strategy framework for pharmaceutical export, it is necessary to develop a pharmaceutical export strategy based on prioritized target markets to develop practical strategies for exporters and the country`s business planning authorities.

Proper management of the pharmaceutical industry can earn huge revenue through pharmaceutical export. The present findings provide useful information that can be crucial for pharmaceutical companies and policymakers who are involving in promoting pharmaceutical export in Iran.
